# Nationwide epidemiological study of subarachnoid hemorrhage: trends in admissions, mortality, seasonality, costs, clipping, embolization, and the impact of COVID-19

**DOI:** 10.3389/fneur.2025.1630224

**Published:** 2025-10-23

**Authors:** Thiago Oscar Goulart, Thire Baggio Machado Marazzi, Rosane Aparecida Monteiro, Gleici da Silva Castro Perdoná, Millene Rodrigues Camilo, Octávio Marques Pontes-Neto

**Affiliations:** ^1^Harvard T.H. Chan School of Public Health, Boston, MA, United States; ^2^Department of Neuroscience and Behavioral Sciences, Ribeirão Preto Medical School, University of São Paulo, Ribeirão Preto, Brazil; ^3^Department of Social Medicine, Ribeirão Preto Medical School, University of São Paulo, Ribeirão Preto, Brazil

**Keywords:** subarachnoid hemorrhage, epidemiology, aneurysms, endovascular treatment, healthcare costs, COVID-19, stroke, neurosurgical clipping

## Abstract

**Introduction:**

Aneurysmal Subarachnoid Hemorrhage (aSAH) represents a severe neurological emergency with high morbidity and mortality. Despite advances in neurocritical care and endovascular techniques, aSAH remains a significant burden, especially in resource-limited settings like Brazil, where studies of this condition are scarce until now. This study evaluates the epidemiology, costs, and procedural trends of aSAH in Brazil from 2017 to 2022, highlighting the impacts of the COVID-19 pandemic.

**Methods:**

This retrospective study analyzed secondary data from the Brazilian public health system (DataSUS) using ICD-10 code I60 for aSAH. Key metrics included the evaluation of admissions with time-series in Python, and mortality rates, procedures, and costs.

**Results:**

Between 2017 and 2022, 61,134 aSAH admissions were recorded, averaging 10,189 annually. The in-hospital mortality rate remained consistently high at 20.3% (range: 19.2–20.5%), with 12,192 total deaths. Admissions declined temporarily during the initial of Pandemic (April–June 2020) but later recovered. Seasonal admission peaks were observed in mid-year months (June–August), while December–February consistently showed lower rates. The average length of hospital stay was 10.0 days, with a decline during the pandemic (9.6 days in 2020) before stabilizing post-pandemic. This pattern likely reflects pandemic-related disruptions and early discharges to manage hospital bed shortages. Procedures for ruptured aneurysms included 8,290 embolizations (13.8%) and 3,043 neurosurgical clippings (5.0%), treating 18.8% of admissions. During the pandemic, procedural volumes declined, with faster recovery in embolizations compared to clippings. Total hospitalization costs were BRL 397,665,436.57 (US$108 million), with an annual increase from BRL 62 million in 2017 to BRL 71 million in 2022. Costs per patient were significantly higher for aSAH compared to other cerebrovascular conditions, reflecting the complexity of care.

**Conclusion:**

This comprehensive analysis highlights the clinical and economic challenges of managing aSAH in Brazil. While admissions and mortality rates remained stable, the pandemic disrupted care delivery and procedural volumes. Low rates of aneurysms secured underscore disparities in access across Brazil. Rising costs emphasize the need for investments in preventive measures, equitable treatment access, and healthcare system resilience. Future strategies should focus on expanding stroke unit coverage and addressing modifiable risk factors to reduce the burden of aSAH.

## Introduction

Aneurismatic Subarachnoid Hemorrhage (aSAH) is a rare type of Stroke, with high social impact worldwide (1). aSAH persists with high morbimortality and poor functional outcome, even with the advances in the neurocritical care, development of guidelines and improvement of devices for the surgical and endovascular treatment of the ruptured aneurysm (2, 3). Nevertheless, the mean costs with treatments are expensive (4, 5), heterogeneous among different countries and crescent in the last years (6, 7). In the face of that, aSAH represents a great burden for the healthcare system, especially in those with limited resources, such as public health systems of underdeveloped countries.

Despite its clinical relevance, epidemiological data on aSAH and its treatment remain scarce in Brazil. In 2007, Minelli et al. published a population-based study conducted in the city of Matão, which recruited 147 stroke patients to evaluate incidence and prognosis. However, only one patient with aSAH was included (8). In 2009, Cabral et al. conducted a population-based study in Joinville, which included 1,353 stroke patients, 55 of whom had aSAH, and also reporting a 6-month mortality rate of 45.5% (95% CI: 36.0–67.6) (9). In 2021, Kurz et al. published data from a Brazilian multicenter cohort of aSAH, encompassing 997 patients admitted to 45 ICUs (10). Approximately 43% of the patients were classified as poor grade (WFNS 4–5), with an in-hospital mortality rate of 34%. The mean duration of hospitalization was 21.3 days (SD: 4.2) for survivors and 13.8 days (SD: 1.3) for non-survivors (10).

In the largest Brazilian prospective multicenter study, which included 471 patients, poor outcomes at discharge (mRS 4–6) were observed in 55% of patients, with long-term poor outcomes (6 months) in 40%. The length of stay (LOS) was reported as 14.5 days (IQR: 7–29) (11). Notably, these studies were conducted in specialized high-volume centers located in more developed regions of Brazil. Consequently, it is plausible that mortality and poor outcomes in Brazil’s national public healthcare system may be higher. It is estimated that three-quarters of the Brazilian population rely exclusively on public healthcare services (12).

Regarding costs, it is estimated that the direct hospital costs per patient in the United States are $82,514; in Germany, €22,470; and in Switzerland, €33,200 (13, 14). In Brazil, private hospitals report costs of approximately $28,928 per patient, whereas public hospitals estimate costs at about $8,031 per patient (15). Sarmento et al. (16) previously studied patients treated by SUS (the Brazilian public health system) between 2009 and 2018. The study found that the mean total hospital costs paid by SUS for aneurysmal disease were approximately $977 for embolization and $221 for surgical clipping, highlighting significant underpayment.

## Objectives

This study analyzes the epidemiology, treatment patterns, costs, and COVID-19 impact on aSAH hospitalizations in Brazil (2017–2022). It evaluates admission trends, in-hospital mortality, aneurysm treatment rates (embolization vs. clipping), healthcare costs, and pandemic-related care disruptions using nationwide DataSUS data.

## Methods

This retrospective epidemiological study analyzed aneurysmal subarachnoid hemorrhage (aSAH, ICD-10 I60) hospitalizations in Brazil from January 2017 to December 2022 using DataSUS, the national health information system. DataSUS aggregates hospital records from the Sistema de Informações Hospitalares (SIH/SUS), covering approximately 75% of hospital admissions in Brazil, including public hospitals and private facilities contracted by the public healthcare system (SUS). The database provides demographic characteristics, hospitalization details, LOS, procedures performed, in-hospital outcomes, and healthcare costs.

aSAH hospitalizations were analyzed monthly to assess temporal trends across pre-, intra-, and post-COVID-19 periods. Yearly trends in mortality rates, procedural volumes (embolization vs. clipping), LOS, and costs were evaluated. Costs, originally in Brazilian Reais (BRL), were converted to USD using annual exchange rates (e.g., 3.19 BRL/USD in 2017 to 5.16 BRL/USD in 2022).

Descriptive and inferential statistical analyses were conducted using Python. To analyze the time series of aSAH admissions, we applied Seasonal-Trend Decomposition using Loess (STL), which allowed us to isolate long-term trends, seasonal variations, and irregular fluctuations in hospitalization patterns.

To assess the impact of the COVID-19 pandemic, we performed a Chow test to detect structural breaks in the trend. The test was applied to March 2020, coinciding with the implementation of lockdown measures, to determine whether there was a statistically significant shift in aSAH admissions.

The primary cohort was defined exclusively based on hospitalizations coded with ICD-10 I60, which corresponds to non-traumatic subarachnoid hemorrhage. To account for potential misclassification in administrative coding practices, we conducted a broader search across all stroke-related ICD-10 codes (I60–I64), including intracerebral hemorrhage (I61), other non-traumatic hemorrhagic strokes (I62), ischemic stroke (I63), and unspecified stroke (I64). This broader search was used to perform a descriptive sensitivity analysis to estimate the proportion of aSAH admissions (ICD-10 I60) among all stroke-related hospitalizations, and to evaluate potential underascertainment due to coding heterogeneity.

To complement the analysis and assess treatment patterns, we identified all occurrences of aneurysm occlusion procedures (clipping or embolization) recorded in the dataset, regardless of the associated diagnosis code. These procedures were not used to define the primary cohort but served to quantify treatment volumes within the I60 group and to detect whether such interventions were also performed in hospitalizations coded under other stroke categories (I61–I64), which might suggest misclassification.

The following SUS billing procedure codes were used to identify aneurysm occlusion treatments:

- Endovascular embolization:

◦ 0403070155 – Aneurysm <1.5 cm with narrow neck
◦ 0403070163 – Aneurysm <1.5 cm with wide neck
◦ 0403070040 – Aneurysm >1.5 cm with narrow neck
◦ 0403070058 – Aneurysm >1.5 cm with wide neck

Microsurgical clipping:

◦ 0403040094 – Anterior circulation >1.5 cm
◦ 0403040108 – Posterior circulation >1.5 cm
◦ 0403040116 – Anterior circulation <1.5 cm
◦ 0403040124 – Posterior circulation <1.5 cm

These procedure codes are fully listed in Supplementary Table S2.

Cases of traumatic subarachnoid hemorrhage were excluded, as they are coded separately under trauma-related ICD-10 categories (e.g., S06.6). Only non-traumatic cases (I60) were included in the primary analysis.

## Results

### Admissions

**Figure 1:**
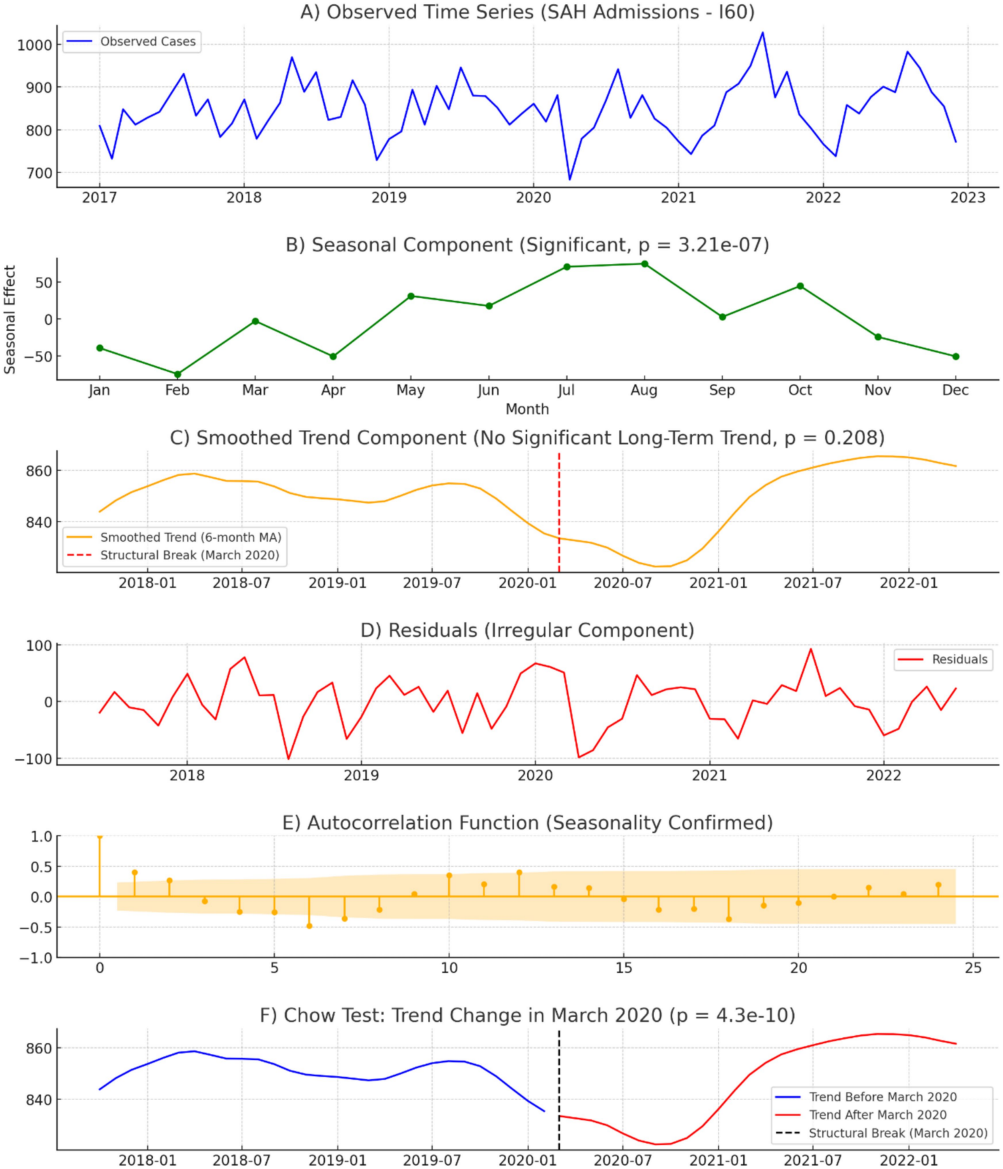
Time series analysis of monthly aSAH Admissions (2017–2022). **(A)** Observed aSAH admissions: monthly hospitalization counts from 2017 to 2022, illustrating a stable pattern before 2020, a sharp decline in early 2020, and a gradual recovery post-pandemic. **(B)** Seasonal component: estimated seasonal variations in aSAH admissions, showing a recurring annual pattern with peak hospitalizations occurring between June and August (winter in Brazil). The seasonal effect was statistically significant (*p* = 3.21 × 10^−7^) **(C)** Smoothed trend component: long-term hospitalization trend using a 6-month moving average. A structural break in March 2020 (red dashed line) marks a significant shift in admissions. The overall trend showed no significant long-term increase or decrease (*p* = 0.208). **(D)** Residuals (irregular component): unexplained variations in aSAH admissions after accounting for trend and seasonality. The most pronounced deviations occurred in April–June 2020, with a sharp decline, followed by an excess of admissions in July 2021, suggesting a compensatory rebound. **(E)** Autocorrelation function (ACF): Confirmed seasonality with significant autocorrelation at 12-month intervals, indicating a persistent annual cycle in aSAH admissions. **(F)** Chow test – trend change in March 2020: Structural break analysis of aSAH admissions before and after the COVID-19 lockdown (March 2020). The chow test detected a statistically significant shift in trend (*p* = 4.3 × 10^−10^), confirming that the pandemic had a measurable impact on hospitalization patterns. This figure illustrates the epidemiological impact of the COVID-19 pandemic on aSAH hospitalizations, highlighting seasonality, trend disruptions, and statistical evidence of a structural break in March 2020.

**Figure 2:**
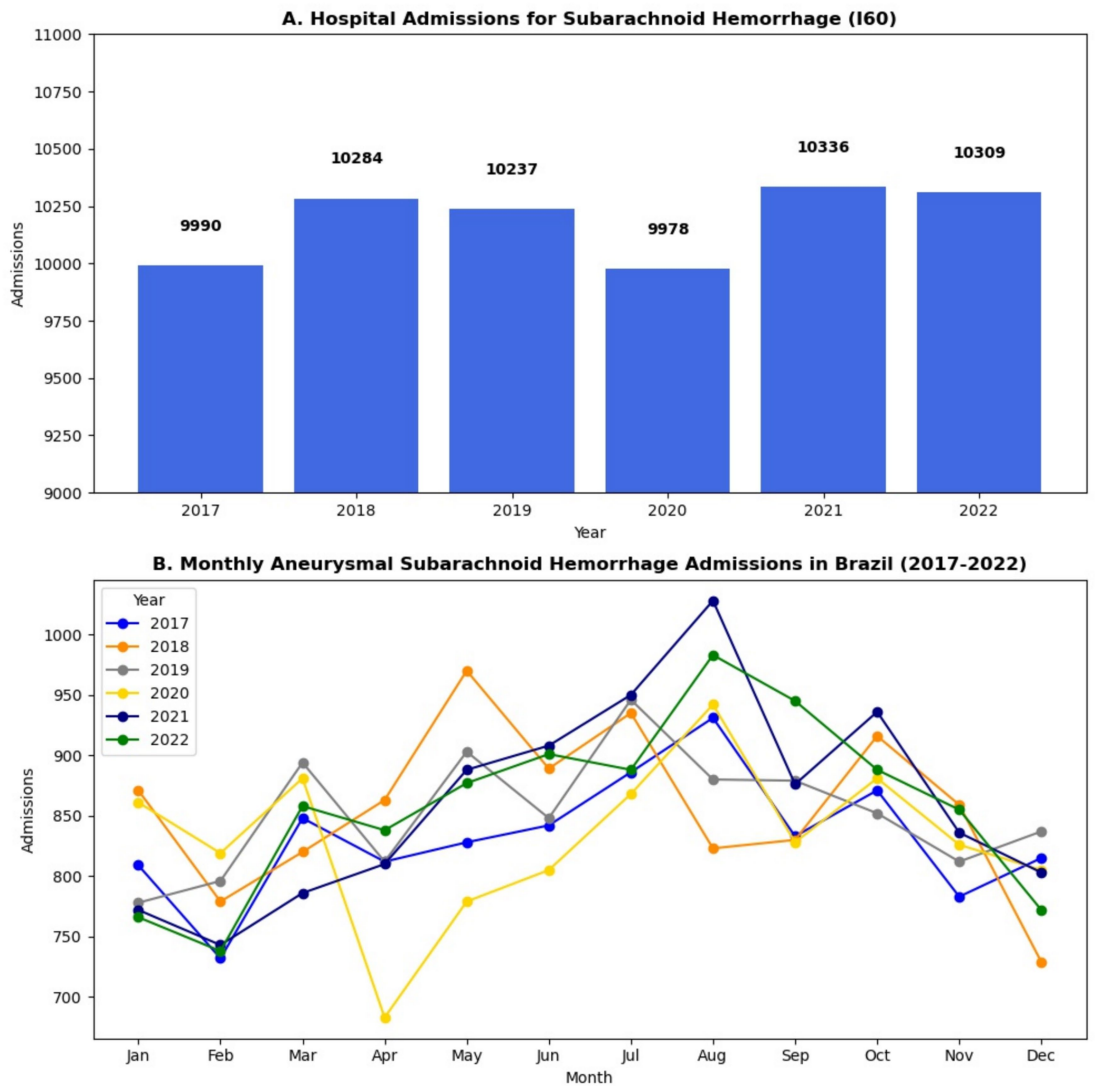
**(A)** Hospital Admissions for aneurismatic Subarachnoid in Brazil (2017–2022). **(B)** Monthly aSAH admission in Brazil (2017–2022).

From January 2017 to December 2022, a total of 61,134 hospitalizations for subarachnoid hemorrhage (non-traumatic aSAH, identified using ICD-10 code I60) were recorded in Brazil. The monthly average number of aSAH admissions was 848.1. Admissions peaked in 2021, with 10,336 cases, while the lowest annual count occurred in 2020, with 9,978 admissions, coinciding with the first year of the COVID-19 pandemic. Figures 1, 2 show that monthly admissions exhibited fluctuations over time, with a sharp decline in April 2020, likely reflecting the impact of hospital restrictions and delayed healthcare-seeking behaviors during the early months of the pandemic.

The trend analysis revealed a relatively stable trajectory in aSAH admissions from 2017 to early 2020, followed by a significant drop in admissions starting in March 2020, coinciding with the implementation of COVID-19 lockdown measures in Brazil. The Chow test confirmed a statistically significant structural break in March 2020 (*p* < 0.001), indicating that the pandemic had a measurable impact on hospitalization rates. Compared to 2019, aSAH hospitalizations declined by 4.3% in 2020, with the most pronounced drop occurring between April and June 2020. A gradual recovery was observed in 2021 and 2022, with admissions returning to pre-pandemic levels by mid-2021 and stabilizing thereafter. The mean trend component was 849.0 ± 42.1 admissions per month, showing moderate long-term fluctuations in hospitalization volumes, as shown in Figure 1.

The seasonal analysis highlighted a recurring annual pattern, with aSAH admissions peaking between June and August, corresponding to the winter months in Brazil. The seasonal amplitude, calculated as the difference between the maximum and minimum seasonal effects, was 112 admissions, indicating a moderate seasonal effect on aSAH hospitalizations. The highest seasonal influence was observed in July, consistently marking the peak in aSAH admissions across multiple years. Despite the pandemic-related disruptions in 2020, the seasonal pattern remained intact, suggesting that environmental and physiological factors associated with colder temperatures contribute to an increased incidence of aSAH. The median seasonal effect was 7.3 admissions, while the Shapiro–Wilk test (*p* = 0.647) indicated that the seasonal component follows a normal distribution, as shown in Figure 1.

The residual analysis identified irregular fluctuations not explained by trend or seasonality. The most pronounced deviations occurred during the early COVID-19 pandemic in 2020, particularly between April and June, when hospital admissions for aSAH experienced a sudden and sharp drop.

The residuals reached a minimum of −78 admissions in April 2020, while the highest deviation occurred in July 2021, with an excess of +92 admissions, suggesting a possible compensatory rebound in hospitalizations post-pandemic. The median residual value was 8.9 admissions, with a mean of −0.16 ± 42.1 admissions. The Shapiro–Wilk test (*p* = 0.0032) confirmed a non-normal distribution of residuals, reinforcing that aSAH admission patterns were significantly disrupted by external factors during the study period.

As a sensitivity analysis, we also evaluated the total number of hospitalizations coded under ICD-10 I60–I64 (all stroke-related admissions), which totaled 1,283,435 during the same period. Among these, I60-coded aSAH represented 4.76%, a proportion consistent with epidemiological estimates reported in other middle- and high-income countries. This supports the specificity of our cohort while acknowledging potential underascertainment due to diagnostic heterogeneity in administrative data.

### Mortality

**Figure 3:**
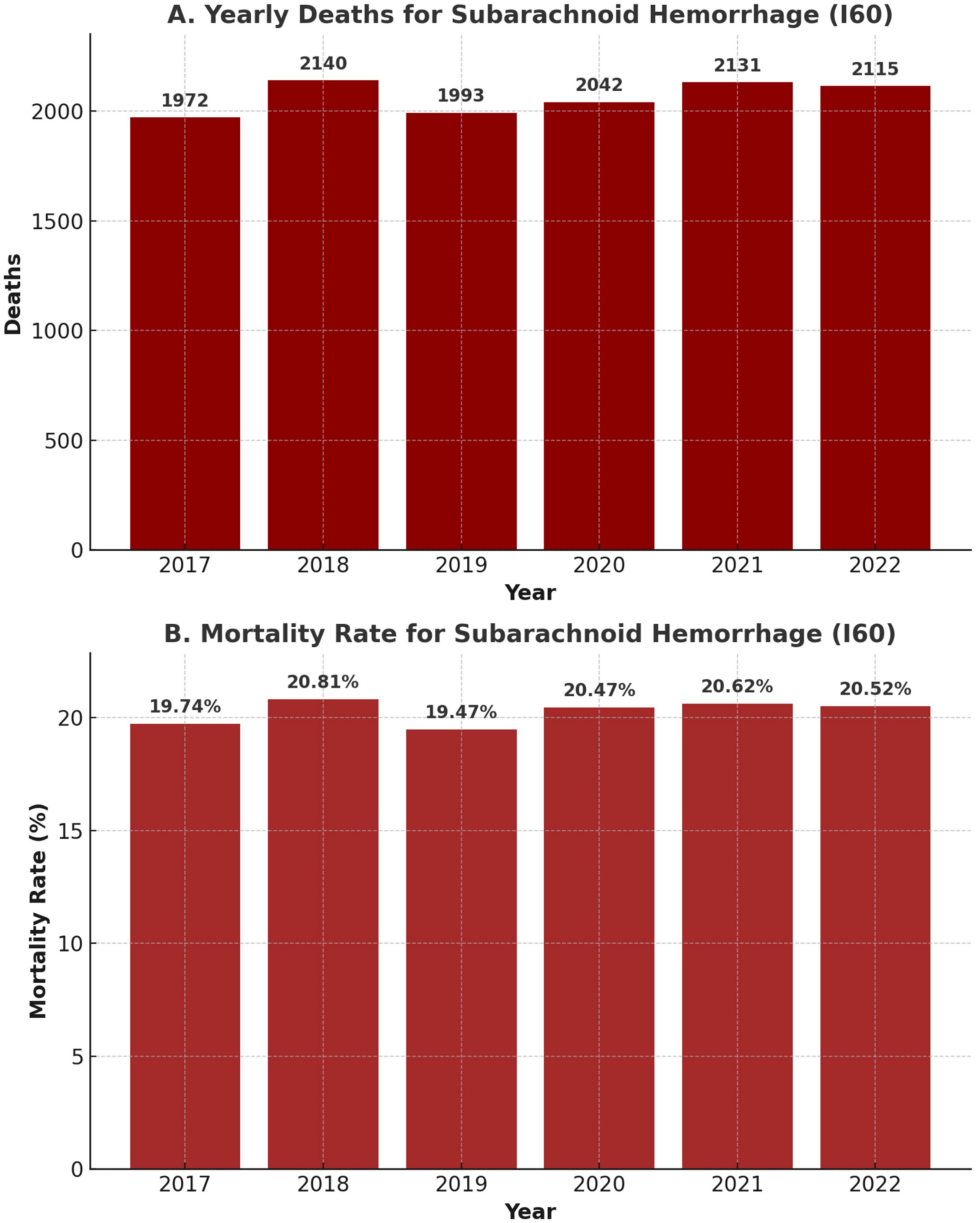
Yearly number of deaths **(A)** and mortality rate **(B)** for aSAH patients (2017–2022).

Figure 3 illustrates the yearly trends in the number of deaths (Panel A) and the mortality rate (Panel B) from 2017 to 2022. Panel A shows a steady increase in the annual number of deaths, rising from 1,972 in 2017 to 2,115 in 2022, while Panel B highlights that the mortality rate remained relatively stable, fluctuating between 19.47 and 20.81% during this period. These trends suggest a growing burden on aSAH while maintaining a consistently, considerably high rate of in-hospital mortality.

### Length of stay

**Figure 4:**
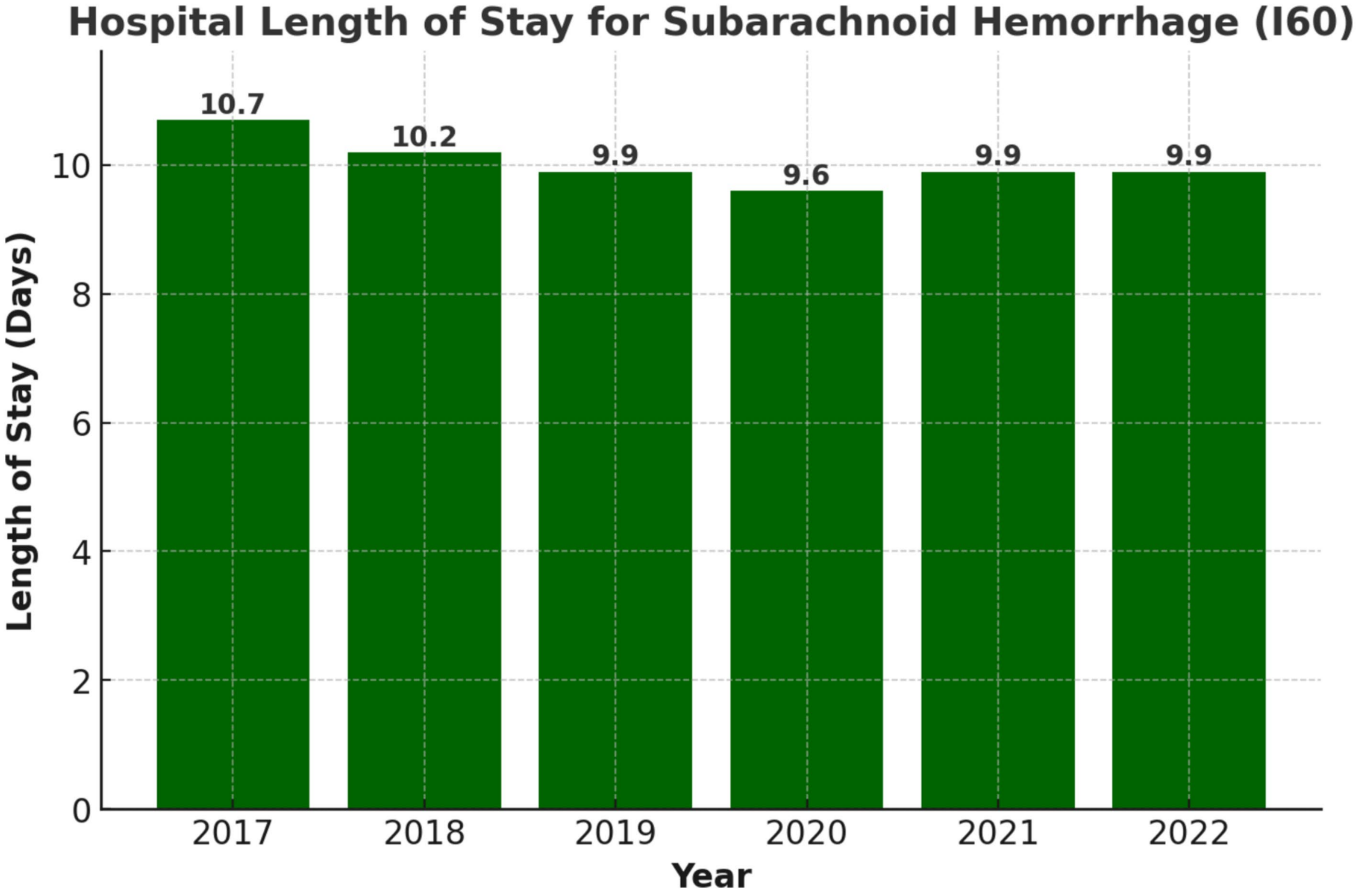
Yearly trends in length of hospital stay (days) for aSAH patients (2017–2022).

The average length of hospital stay for aSAH patients was 10.0 days across the entire period, with year-by-year variations, as shown in Figure 4. In 2017, the average LOS as the longest at 10.7 days, gradually declining to 9.6 days in 2020 during the COVID-19 pandemic. The hospitalization duration increased slightly to 9.9 days in 2021 and remained consistent in 2022. Prolonged stays were associated with complications such as delayed cerebral ischemia, hydrocephalus requiring cerebrospinal fluid diversion, or infections. The total LOS for all patients during the study period amounted to 613,233 patient-days.

### Procedures

**Figure 5:**
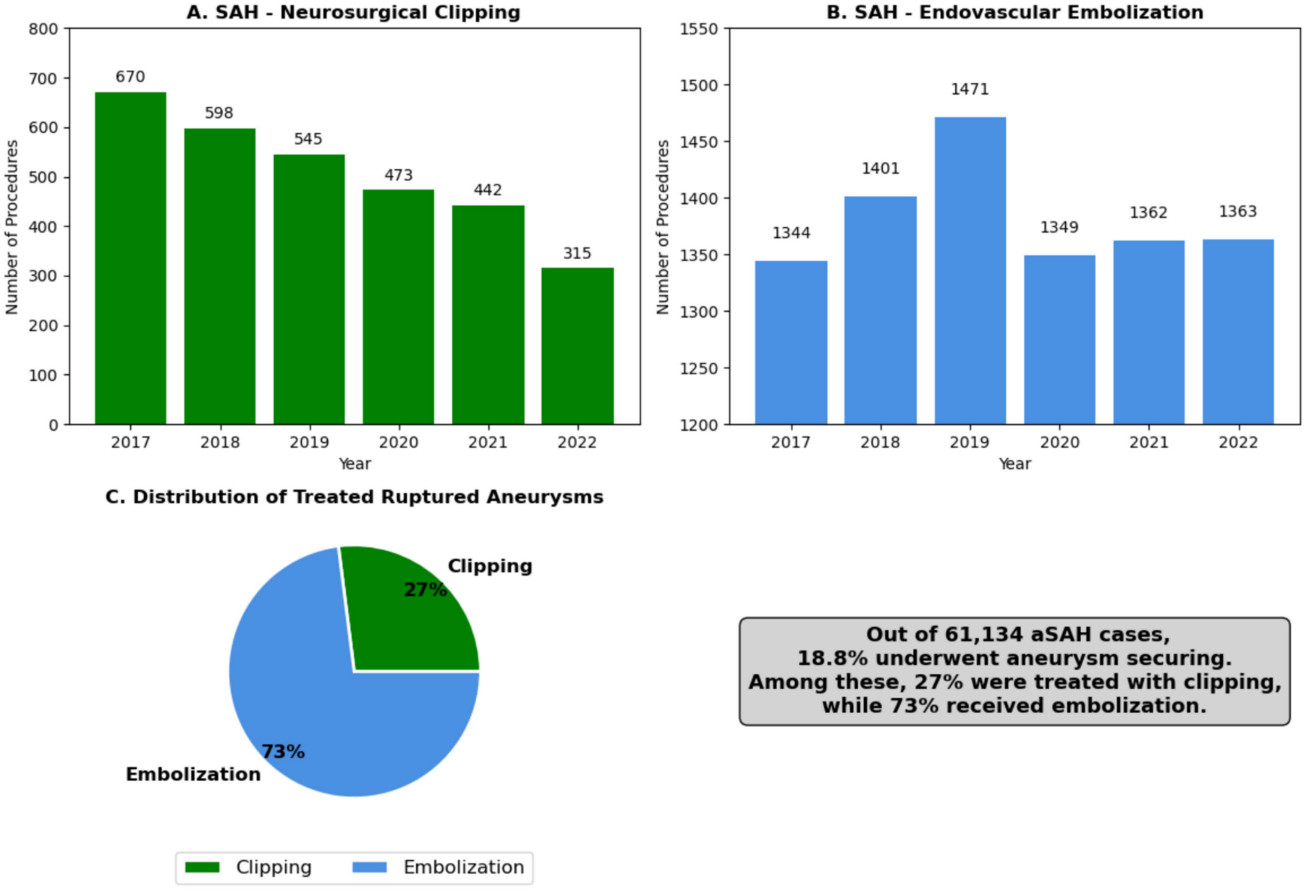
Trends in treatment modalities for aneurismatic subarachnoid hemorrhage (2017–2022): Neurosurgical clipping vs. Endovascular embolization: **(A)** Yearly number of neurosurgical clippings in Brazil (2017–2022), **(B)** Yearly number of endovascular embolization in Brazil (2017–2022), **(C)** Number of ruptured aneurysms treated and percentual comparison between clipping and embolization.

Treatment modalities for aSAH have evolved, with a growing preference for endovascular techniques. A total of 8,290 embolizations were performed during the study period, accounting for 73.37% of treated aneurysms. Neurosurgical clipping was performed in 3,043 cases, comprising 26.63% of treatments (shown in Figure 5). This shift toward embolization aligns with global trends favoring minimally invasive approaches for managing ruptured aneurysms.

The COVID-19 pandemic caused a notable decline in procedures. Embolizations decreased by 9.4%, and neurosurgical clippings declined by 15.2% in 2020 compared to 2019. Procedural volumes began recovering in 2021, with embolizations showing a faster rebound compared to surgical clipping (shown in Figure 5).

**Figure 6:**
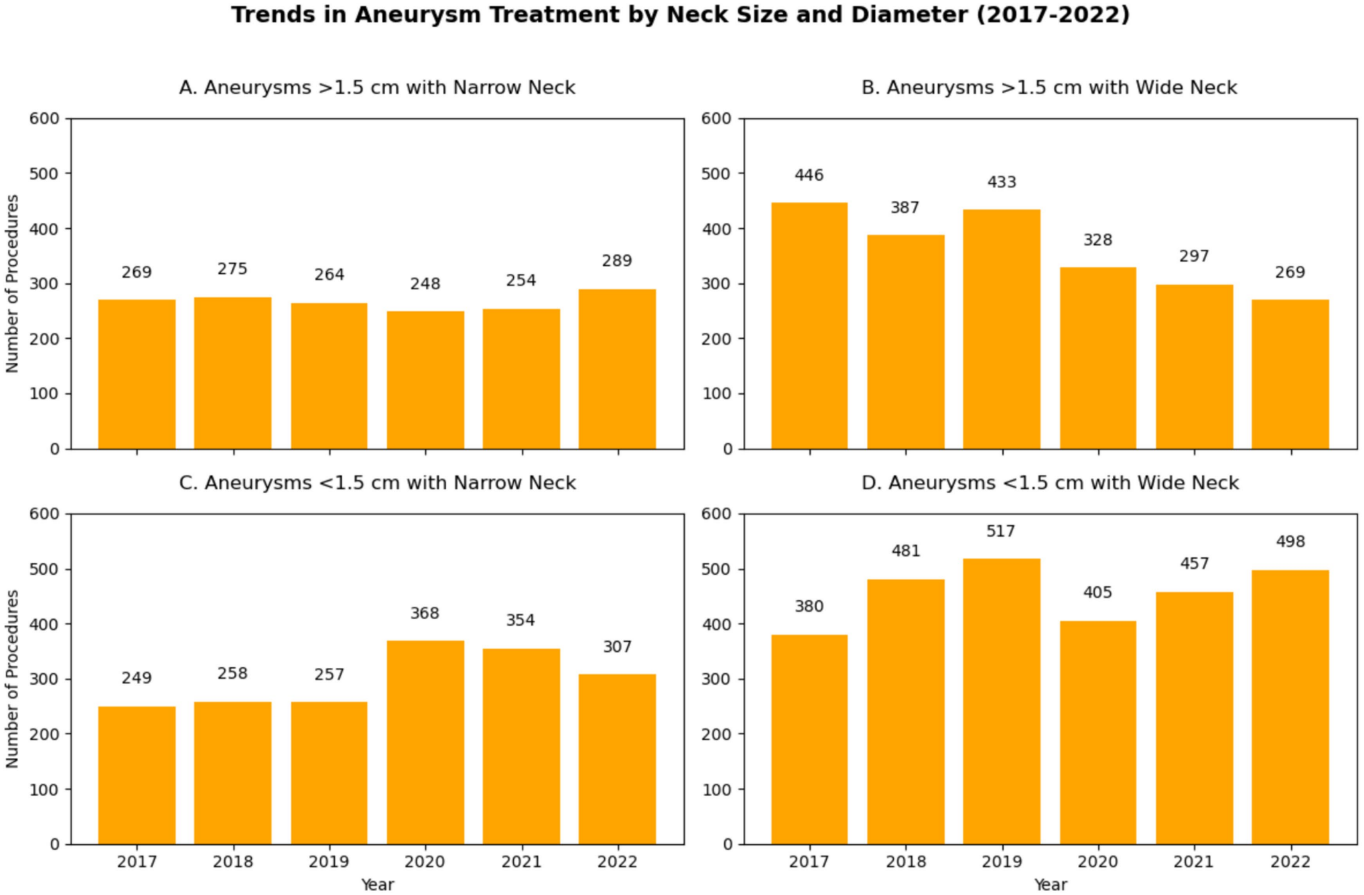
Embolization of intracranial aneurysms. **(A)** Aneurysms with size less than 1.5 cm and with Narrow Neck; **(B)** Aneurysms measuring more than 1.5 cm with Wide Neck; **(C)** Aneurysms less than 1.5 cm with Narrow Neck; **(D)** Aneurysms less than 1.5 cm with Wide Neck.

**Figure 7:**
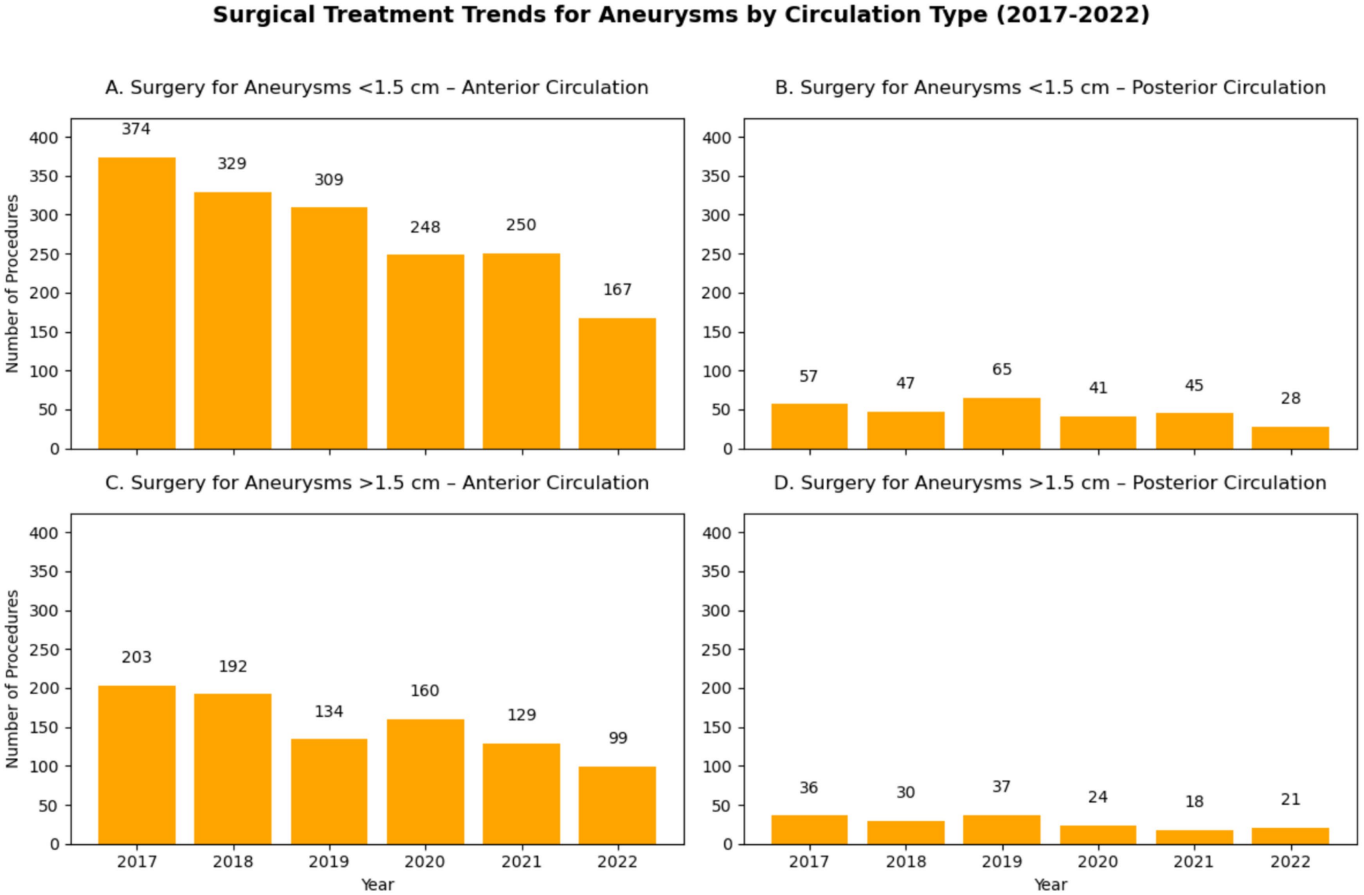
Surgical Clipping of intracranial aneurysms: **(A)** Aneurysms less than 1.5 cm from Anterior Circulation; **(B)** Aneurysms less than 1.5 cm from Posterior Circulation; **(C)** Aneurysms more than 1.5 cm from Anterior Circulation; **(D)** Aneurysms more than 1.5 cm from Posterior Circulation.

Figures 6, 7 show that narrow-neck aneurysms >1.5 cm saw a moderate increase, while wide-neck aneurysms declined significantly. Smaller aneurysms (<1.5 cm) remained stable, with a slight increase in wide-neck cases. Anterior circulation aneurysm surgeries declined sharply, while posterior circulation procedures showed a steady decrease over time.

### Costs

**Figure 8:**
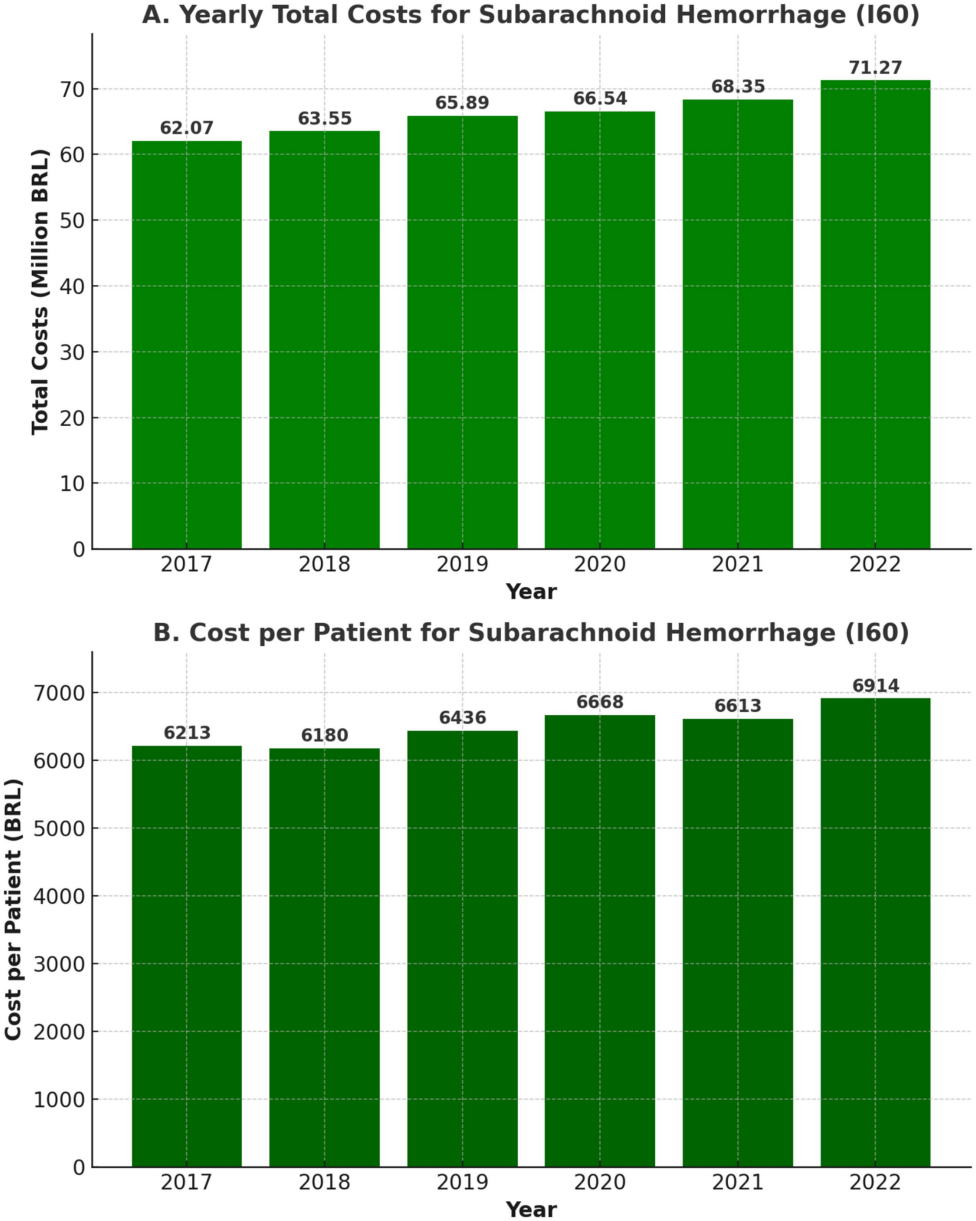
Yearly costs in reais **(A)** and yearly costs per patient in reais **(B)** for aSAH Patients (2017–2022).

The financial burden of aSAH on Brazil’s healthcare system was significant. Total hospitalization costs over the six years amounted to R$397,665,435.57, equivalent to approximately US$108 million. Annual costs increased steadily, from R$62,066,579.92 (US$19.5 million) in 2017 to R$71,273,543.82 (US$13.7 million) in 2022, driven by the adoption of advanced treatments and inflationary pressures (shown in Figure 8A).

The average cost per hospitalization was R$6,504.82 (US$1,895), as shown in Figure 8B, significantly higher than for other cerebrovascular conditions, such as ischemic stroke (R$1,965.58 or US$572) or intracerebral hemorrhage (R$3,378.85 or US$984).

## Discussion

This study provides a comprehensive epidemiological analysis of aSAH hospitalizations in Brazil from 2017 to 2022, highlighting trends in admissions, mortality, LOS, treatment approaches, and healthcare costs. The findings reveal consistent seasonal variations, with a significant impact of the COVID-19 pandemic on aSAH hospital care. Also, there was an ongoing shift toward endovascular management of aneurysmal aSAH, mirroring global trends in cerebrovascular disease treatment.

### aSAH admissions, seasonal patterns, and the impact of COVID-19

In Brazil, aSAH accounts for 4.76% of all stroke-related hospitalizations, a proportion consistent with global trends, where aSAH represents a smaller but clinically significant component of cerebrovascular diseases requiring intensive care (17). The findings of this study highlight the substantial burden of aSAH hospitalizations, with 61,134 cases recorded from 2017 to 2022, averaging 10,189 admissions per year.

Although annual hospitalization rates remained relatively stable, a clear seasonal pattern emerged, with higher admission rates between June and August (winter months) and lower rates in December (shown in Figures 1, 2). This trend is consistent with global data, where colder temperatures are associated with increased blood pressure variability, vasoconstriction, and a heightened risk of aneurysm rupture (18). The highest single-month admission count occurred in August 2021 (1,028 cases), whereas December consistently recorded the lowest hospitalization numbers, potentially reflecting seasonal variations in healthcare-seeking behavior (18).

Despite this overall stability, a notable decline in aSAH admissions occurred in 2020, particularly from April to June, coinciding with the first wave of the COVID-19 pandemic. Compared to 2019, hospitalizations declined by 4.3% in 2020, marking the lowest annual count in study period. This drop may reflect underreporting, delays in seeking emergency care, and disruptions in hospital capacity, but more importantly, it likely signifies an increase in pre-hospital mortality, as aSAH has high fatality rates before hospital arrival. Following this temporary decline, admission rates rebounded in 2021, indicating that hospitals adapted to pandemic-related constraints, restoring access to aSAH care.

### Mortality trends and length of stay

aSAH remains a highly fatal condition, with in-hospital mortality rates ranging from 19.47 to 20.81% over the study period (as shown in Figure 3). The absolute number of deaths increased annually, rising from 1,972 in 2017 to 2,115 in 2022, reflecting the growing burden of aSAH on Brazil’s healthcare system. However, these figures only account for in-hospital mortality, meaning they do not capture pre-hospital deaths, which are particularly relevant given the drop in hospital admissions in 2020.

Although there is relative consistency in the global literature regarding the reduction in case fatality from aSAH in population-based studies, there is still limited data on the reduction of in-hospital mortality (19). The analyzed rates for clinical comparison were extracted from studies published about a decade ago in the U. S. and Canada. They reported rates between 18 and 21.5%, similar to those found in this analysis of DATASUS data.

However, it is important to note that when examining in-hospital mortality rates for aSAH across all public hospitals in Brazil, performance appears comparable to that of high-tech centers with advanced clinical specialization and high case volumes, particularly in the southeastern region of Brazil (10). The authors of this study propose several hypotheses to explain these findings. First, that DATASUS does not allow for the assessment of the severity of patients admitted to each center, hindering comparisons with similar populations. Second, that reporting cases of aSAH is not mandatory in Brazil and tends to occur primarily in referral centers. And lastly, we believe that the rate of patients who die from aSAH without a clinical diagnosis in the country is likely higher, leading to an underreporting of in-hospital mortality rates for this condition.

The average LOS for aSAH patients ranged from 9.6 to 10.7 days over the years (shown in Figure 4), with a decline in 2020 likely due to ICU bed shortages and early discharges during the pandemic. This stability may reflect high pre-hospital mortality among severe cases and early in-hospital deaths, rather than improvements in care.

The short average LOS (<14 days) suggests many patients may not receive full transcranial Doppler (TCD) monitoring for vasospasm, a key secondary injury risk (20). While TCD has not been directly linked to reduced mortality, its limited availability in Brazil, with only 250 certified stroke units as of 2022, underscores the need for expanded neurocritical care training and hospital infrastructure to improve aSAH management (20–22).

### Endovascular embolization

Endovascular embolization became the dominant treatment modality for ruptured aneurysms, with 8,290 embolizations performed, representing 73.37% of treated aneurysms, as shown in Figure 5. This trend aligns with global practices favoring minimally invasive approaches, which are associated with lower morbidity, reduced hospital stays and improved long-term outcome.

However, the COVID-19 pandemic led to a 9.4% decline in embolization procedures in 2020, reflecting operating room restrictions, ICU shortages, and prioritization of COVID-19 cases over neurovascular interventions. Despite this setback, embolization rates recovered by 2021, reinforcing the growing role of endovascular treatment in aSAH management.

Moreover, as highlighted by Danière et al., complications such as vasospasm, acute hydrocephalus, ischemic injury, and delayed cerebral infarction remain major determinants of morbidity and mortality after aneurysmal subarachnoid hemorrhage, even in patients successfully treated by endovascular embolization (23). The incidence and impact of these events vary among healthcare systems, emphasizing the importance of post-procedural monitoring and long-term follow-up strategies to mitigate late adverse outcome.

### Neurosurgical clipping

Neurosurgical clipping remains a critical treatment option, particularly for aneurysms that are not amenable to endovascular techniques. However, only 3,043 cases (26.63% of treated aneurysms) were managed with clipping. This lower rate may be attributed to the limited availability of specialized neurovascular treatment centers, despite the broader accessibility of neurosurgical procedures in Brazil. More likely, it reflects the growing preference for endovascular treatment, which has shown an upward trend over the study period, as shown in Figure 5.

The pandemic had a more pronounced impact on clipping than on embolization, with a 15.2% decline in 2020, reflecting greater procedural delays and ICU resource constraints. Furthermore, analysis of treatment patterns by aneurysm location and size (shown in Figures 6, 7) revealed that clipping rates for anterior circulation aneurysms significantly decreased, while both surgical and endovascular procedures for posterior circulation aneurysms showed a gradual decline.

These trends highlight ongoing disparities in aneurysm treatment access, with only 18.8% of ruptured aneurysms in Brazil receiving definitive treatment. Expanding endovascular training, improving surgical capacity, and addressing geographic inequalities in neurovascular care remain critical priorities.

### Economic burden of aSAH and cost trends

aSAH imposes a significant financial burden on Brazil’s healthcare system, with total hospitalization costs exceeding R$397 million (US$108 million) over six years, as shown in Figure 8A. The average cost per hospitalization was R$6,504.82 (US$1,895), substantially higher than ischemic stroke (R$1,965.58; USD $572) or intracerebral hemorrhage (R$3,378.85; US$984), reflecting the intensive care requirements and procedural costs associated with aSAH.

Annual costs steadily increased, reaching R$71.2 million (US$13.7 million) in 2022, driven by inflation, procedural expansion, and the rising adoption of endovascular therapy, as shown in Figure 8B. In 2020, costs temporarily declined alongside reduced aSAH admissions and procedures due to the pandemic but rebounded in 2021, highlighting aSAH’s persistent economic burden. While embolization has higher upfront costs, its potential to reduce complications and ICU stays supports continued investment in neurointerventional training and infrastructure.

### Strengths

This nationwide study is one of the largest analyses of aSAH hospitalizations, offering insights into epidemiological trends, procedural shifts, and economic burden over six years. Using DataSUS, it ensures broad Brazilian population coverage while minimizing selection bias.

Time-series decomposition reveals long-term trends, seasonal variations, and COVID-19-related disruptions, providing a framework for healthcare system resilience evaluation, while also quantifying pandemic-driven declines in embolization and clipping rates, highlighting access challenges in a continental-sized middle-income country.

The cost analysis underscores aSAH’s economic burden, reinforcing the need for policy interventions to expand endovascular therapy access, reduce treatment disparities, and optimize resource allocation in Brazil’s public healthcare system.

## Limitations

This study has several limitations. Relying on hospital admission data from DataSUS may introduce coding inconsistencies and misclassification biases. As a sensitivity analysis, we conducted a broader search to identify all hospitalizations coded as stroke (ICD-10 I60–I64) in order to estimate the proportion specifically attributed to aSAH (I60).

Additionally, the low treatment rate observed may reflect a combination of underreporting of procedures, limited neurovascular capacity—particularly in underserved regions—and potential misclassification of diagnoses. Given the limited number of certified stroke units in Brazil, especially outside major urban centers, this structural limitation is likely a key contributor to the underutilization of aneurysm treatment. According to a national survey by Martins et al. (22), there are around 300 stroke units in Brazil, a small figure for a continental-sized country with more than 200 million inhabitants. Also, there are substantial regional disparities in their distribution, as 77% are concentrated in South and Southeast regions (22).

The analysis is based on hospitalization records rather than individual patient data, likely underestimating aSAH incidence due to high pre-hospital mortality while potentially overestimating admissions due to readmissions. Cases treated in private hospitals outside the SUS system were not captured, affecting completeness and generalizability.

Furthermore, key clinical variables—such as aneurysm characteristics, aSAH severity (Hunt-Hess/WFNS), treatment delays, and functional outcomes—were unavailable, limiting assessments of stroke severity and prognosis. Regional disparities in access to neurocritical care and neurointerventional procedures were also not analyzed. Finally, although we included all procedures performed in SUS hospitals, regional disparities in access to neurocritical care and neurointerventional therapies could not be evaluated in this dataset and remain an important topic for future research.

## Conclusion

Despite stable hospitalization and mortality rates, aSAH admissions declined during the first wave of COVID-19, likely due to healthcare access disruptions and increased pre-hospital mortality. Procedural volumes dropped in 2020 but recovered in subsequent years. Seasonal peaks in aSAH hospitalizations (June–August) highlight the role of environmental factors, emphasizing the need for seasonal preparedness in stroke care.

The increasing preference for embolization aligns with global trends, yet the low percentage of treated aneurysms (18.8%) suggests barriers to access, particularly in resource-limited areas. The economic burden of aSAH remains significant, exceeding R$397 million (USD $108 million) over six years.

Efforts should prioritize faster aSAH recognition, expanded access to treatment, and healthcare system resilience. Investments in neurointerventional training, infrastructure, and preventive strategies—such as hypertension control and smoking cessation—are crucial to reducing aSAH incidence and improving outcomes. These findings provide valuable insights for optimizing aSAH care and informing health policy in Brazil.

## Data Availability

The raw data supporting the conclusions of this article will be made available by the authors, without undue reservation.
