# Supplementary material for: Nationwide epidemiological study of subarachnoid hemorrhage: trends in admissions, mortality, seasonality, costs, clipping, embolization, and the impact of COVID-19
Source: Front Neurol. 2025 Oct 23;16:1630224. doi: 10.3389/fneur.2025.1630224 (PMC12590765; doi:10.3389/fneur.2025.1630224)
Supplement: Supplementary file 1 [file Data_Sheet_1.pdf]

Supplementary Table S1. Total Number of Stroke-Related Hospitalizations in Brazil by ICD-10 Code (2017–2022)

ICD-10 Code	Description	Number of Hospitalizations	Proportion of Total (%)
I60	Non-traumatic subarachnoid hemorrhage	61,134	4,76%
I61	Intracerebral hemorrhage	104,420	8,14%
I62	Other nontraumatic intracranial hemorrhage	23,884	1,86%
I63	Cerebral infarction (ischemic stroke)	765,442	59,65%
I64	Stroke, not specified as hemorrhage or infarction	328,555	25,59%
**Total**		1,283,435	100%

Supplementary Table S2. Procedure Codes Used to Identify Aneurysm Treatments in the SUS Database (2017–2022)

Procedure Type	SUS Code	Description
**Endovascular Embolization**	0403070155	Embolization of cerebral aneurysm <1.5 cm with narrow neck
	0403070163	Embolization of cerebral aneurysm <1.5 cm with wide neck
	0403070040	Embolization of cerebral aneurysm >1.5 cm with narrow neck
	0403070058	Embolization of cerebral aneurysm >1.5 cm with wide neck
**Microsurgical Clipping**	0403040094	Microsurgery for anterior circulation aneurysm >1.5 cm
	0403040108	Microsurgery for posterior circulation aneurysm >1.5 cm
	0403040116	Microsurgery for anterior circulation aneurysm <1.5 cm
	0403040124	Microsurgery for posterior circulation aneurysm <1.5 cm
